# Relation between preoperative use of diuretics and renal replacement therapy after cardiac surgery: a propensity score analysis

**DOI:** 10.1186/cc13580

**Published:** 2014-03-17

**Authors:** E Curiel-Balsera, M Delange van de Kroft, I Macias-Guarasa, R Hinojosa Perez, J Ravina-Sanz, M Garcia-Delgado, R Rivera-Fernandez

**Affiliations:** 1 Carlos Haya University Hospital, Malaga, Spain; 2 County Hospital of Axarquia, Velez Malaga, Spain

## Introduction

The aim was to evaluate the relation between preoperative use of diuretics and renal replacement therapy (RRT) in postoperative patients of cardiac surgery.

## Methods

A prospective cohort of adult patients who underwent cardiac surgery in 11 institutions all over the Andalusia community from March 2008 to July 2012 was included in the ARIAM adult cardiac surgery project. Diuretic users prior to the intervention were pair-matched to nonusers on the basis of a propensity score based on demographics, comorbidities, medication and surgical data. We analysed differences in RRT in both groups.

## Results

The total cohort was composed of 7,276 patients with 63.91 ± 12.45 years and 61.1% male gender. Elective scheduled surgery was done in 85.9% of patients. Surgical risk assessed by the additive EuroSCORE was 5.86 ± 3.14 points and predicted mortality by the Logistic EuroSCORE was 8.10%. Mortality in the ICU was 7.6% and inhospital mortality was 10.1% (8.1% missing data). Prior to surgery, 10.4% of patients had a creatinine level between 1.2 and 2 mg/dl, 1.1% between 2 and 2.3 mg/dl, 0.8% between 2.3 and 3.5 mg/dl and 0.3% above 3.5 mg/dl. In the nonmatched cohort, 180 patients (2.5%) needed RRT. RRT was needed in 3.5% of 3,771 patients with diuretics and in 1.4% of 3,505 patients not treated with them (*P *< 0.001); 2.61 (1.87 to 3.65). After adjusting with logistic regression by additive EuroSCORE, SAPS 3, bypass time exceeding 120 minutes and prior renal dysfunction, the OR was 1.67 (1.15 to 2.45). When we analysed 3,426 matched patients according to the propensity score, RRT was needed in 3% of 1,713 patients with diuretics and in 1.6% of 1,713 not treated with them (*P *< 0.009), 1.85 (1.16 to 2.94).

## Conclusion

Preoperative use of diuretics is associated with an increased risk of need for RRT.

